# Tabanidae (Diptera) collected on horses in a Cerrado biome in the state of Tocantins, Brazil

**DOI:** 10.1590/S1984-29612024036

**Published:** 2024-07-15

**Authors:** Mariana Vaz da Costa, Gratchela Dutra Rodrigues, Helena Iris Leite de Lima, Tiago Kütter Krolow, Rodrigo Ferreira Krüger

**Affiliations:** 1 Programa de Pós-graduação em Biodiversidade, Ecologia e Conservação, Universidade Federal do Tocantins – UFT, Porto Nacional, TO, Brasil; 2 Programa de Pós-graduação em Biodiversidade Animal, Universidade Federal de Pelotas – UFPel, Pelotas, RS, Brasil; 3 Programa de Pós-graduação em Entomologia, Universidade Federal de Pelotas – UFPel, Pelotas, RS, Brasil; 4 Universidade Federal do Tocantins – UFT, Porto Nacional, TO, Brasil; 5 Instituto de Biologia, Universidade Federal de Pelotas – UFPel, Pelotas, RS, Brasil

**Keywords:** Horse flies, Cerrado biome, vectors, diversity, Mutucas, bioma Cerrado, vetores, diversidade

## Abstract

Tabanidae (Diptera), popularly known as horse flies, is an important vector group. This is the first study to ascertain the abundance and diversity of horse flies in horses at the cerrado biome of the state of Tocantins, Brazil. Collecting took place in typical Cerrado, and sampling occurred in the dry and rainy seasons. The horseflies were collected from horses using an entomological net. A total of 249 individuals were collected and spread over 25 species. The prevalent species were *Stypommisa aripuana* (25.8%) and *Catachlorops rufescens* (6.4%), in the dry period, and *Fidena lissorhina* (22.5%), *Tabanus occidentalis* var. *dorsovittatus* (10%) and *Poeciloderas quadripunctatus* (6.4%), in the rainy season. The results suggest that tabanids attack horses throughout the dry and rainy seasons, posing a constant threat to their health in the Cerrado of Tocantins.

## Resumo

Os tabanídeos, popularmente conhecidos como mutucas, representam um importante grupo de vetores. Este é o primeiro estudo a verificar a abundância e diversidade de mutucas em cavalos, no bioma cerrado do estado do Tocantins, Brasil. As coletas ocorreram em ambiente típico de Cerrado e as amostragens nos períodos seco e chuvoso. Mutucas foram coletadas de cavalos por meio de rede entomológica. Um total de 249 indivíduos foi coletado, representando 25 espécies. As espécies mais abundantes foram *Stypommisa aripuana* (25,8%) e *Catachlorops rufescens* (6,4%), no período seco, e *Fidena lissorhina* (22,5%), *Tabanus occidentalis* var. *dorsovittatus* (10%) e *Poeciloderas quadripunctatus* (6,4%), no período chuvoso. Os resultados sugerem que os tabanídeos atacam equinos ao longo das estações seca e chuvosa, constituindo uma ameaça constante à sua saúde no Cerrado do Tocantins.

## Introduction

Tabanidae flies are present all over the globe, except in Antarctica. Males have a nectarivorous feeding habit, whereas females are hematophagous, seeking blood meals in mammals, birds, amphibians and reptiles. Females need blood for the maturation of developing oocytes (Fairchild, 1981; Foil & Hogsette, 1994).

Due to the blood-sucking habit of females, horse flies are important to animal health. They are biological and mechanical vectors of pathogens that affect farmed, domestic and wild animals across the globe (Krinsky, 1976; Foil, 1989). Among the pathogens associated with horse flies, in Brazil, 20 of them that cause diseases such as anthrax, tularemia and anaplasmosis, among various forms of trypanosomiasis and filariasis (Baldacchino et al., 2014).

It is estimated that there are more than 4,525 species of tabanids in the world, currently allocated in 177 genera (Bánki et al., 2023). In the Neotropical region, there are 1,205 species (Henriques et al., 2012), and in Brazil alone, 489 species have been recorded (Krolow & Henriques, 2022). The Brazilian state of Tocantins has 65 registered species (Oliveira et al., 2022).

There are approximately 125,000 horses in Tocantins (IBGE, 2017). The primary diseases that affect these animals are equine infectious anemia (EIA), rabies, glanders and vesicular stomatitis (Brasil, 2022), of which only vesicular stomatitis and EIA have been associated with horse flies as vectors (Baldacchino et al., 2014). In Tocantins alone, 4813 cases of AIE were reported between 2005 and 2021 (Brasil, 2022) and may have hematophagous flies as vectors.

Data on equine trypanosomiasis, caused by the protozoan *Trypanosoma evansi* (Steel, 1885) (Kinetoplastida: Trypanosomatidae), have not been collected in Tocantins, but outbreaks of the disease have been observed in neighbouring states: Mato Grosso (Franke et al., 1994), and Pará (Silva et al., 2016).

The abundance of tabanids has been correlated with disease outbreaks that affect livestock. In the Rio Grande do Sul (Rodrigues et al., 2005), an outbreak of equine trypanosomiasis, during the summer coinciding with the tabanid season, victimized dozens of horses. Ecological studies in the same area showed that the activity peak of adult insects occurred in the hottest periods of the year (late spring and summer) and suggested that *Tabanus triangulum* Wiedemann, 1828 was a strong candidate for the mechanical transmission of pathogens in the region, due to its prevalence there (Krüger & Krolow, 2015). Recently, *Trypanosoma evansi* DNA was found in the mouthparts of *Dichelacera alcicornis* (Wiedemann, 1828) and *D. januarii* (Wiedemann, 1819) (Ramos et al., 2023) in the state of Santa Catarina (first record for South America). In the Pantanal biome, Barros (2001) noticed that the rainy season offers the highest risk of mechanical transmission of pathogenic agents by tabanids. Since *T. importunus* (Wiedemann, 1828), was the most abundant species in that region, it was suggested that it is the most likely vector of pathogens to horses to the Pantanal. Equine trypanosomiasis is endemic to the Pantanal, and outbreaks occur concomitantly with peaks of tabanids in the rainy season (Silva et al., 1995; Barros & Foil, 1999; Dávila et al., 2003).

No method is effective to control attacks by tabanids on animals. Even so, identifying activity peaks in the populations of these insects helps to devise strategies to try to reduce tabanid bites on herds. Several studies have been carried out in Brazil to understand the seasonality and the abundance of horse flies biting horses: Eastern Amazon, state of Pará (Gorayeb, 1993, 2000; Luz-Alves et al., 2007); western Amazon, state of Rondônia (Zamarchi et al., 2023); Serrano Plateau, state of Santa Catarina (Miletti et al., 2011); Pantanal, state of Mato Grosso do Sul (Barros, 2001); Brazilian Pampa, Rio Grande do Sul (Krüger & Krolow, 2015); and Uruguayan pampa (Lucas et al., 2020). As for the Cerrado biome, only one study on tabanid seasonality was carried out, in which Koller et al. (2019) collected tabanids with malaise traps in a Pantanal-Cerrado transition area in the state of Mato Grosso do Sul. No study has been carried out on the abundance of tabanids in the Cerrado of Tocantins.

Considering the size of the equine herd in the state of Tocantins and that two of the four diseases registered by the Ministry of Agriculture, which affect equines, may be transmitted by horse flies, the potential economic impact of tabanids should be taken into account. For this reason, it is important to understand how the abundance of tabanids during the year in the Cerrado of Tocantins. This knowledge will uncover the activity patterns of the most abundant species that may play a major role as vectors of pathogens in this region. These patterns can also subsidize prevention and control strategies and contribute to the knowledge of Tabanidae populations in the Cerrado biome.

## Material and Methods

### Study area

The cerrado biome stretches over the states of Bahia, Goiás, Maranhão, Mato Grosso, Mato Grosso do Sul, Minas Gerais, Piauí, Rondônia, São Paulo, Tocantins and the Federal District. It is the second-largest Brazilian vegetation formation, extending over 2 million square kilometers (Ribeiro & Walter, 1998). The seasonal regime of the Cerrado is typically tropical, equivalent to the AW type, with dry winter (from October to April) and rainy summer (from May to September) (Silva et al., 2008). In some areas of Cerrado, as in the states of Goiás, Mato Grosso, Minas Gerais, Bahia and Tocantins, water is deficient in five to six months of the year (Silva et al., 2008).

**Figure 1:**
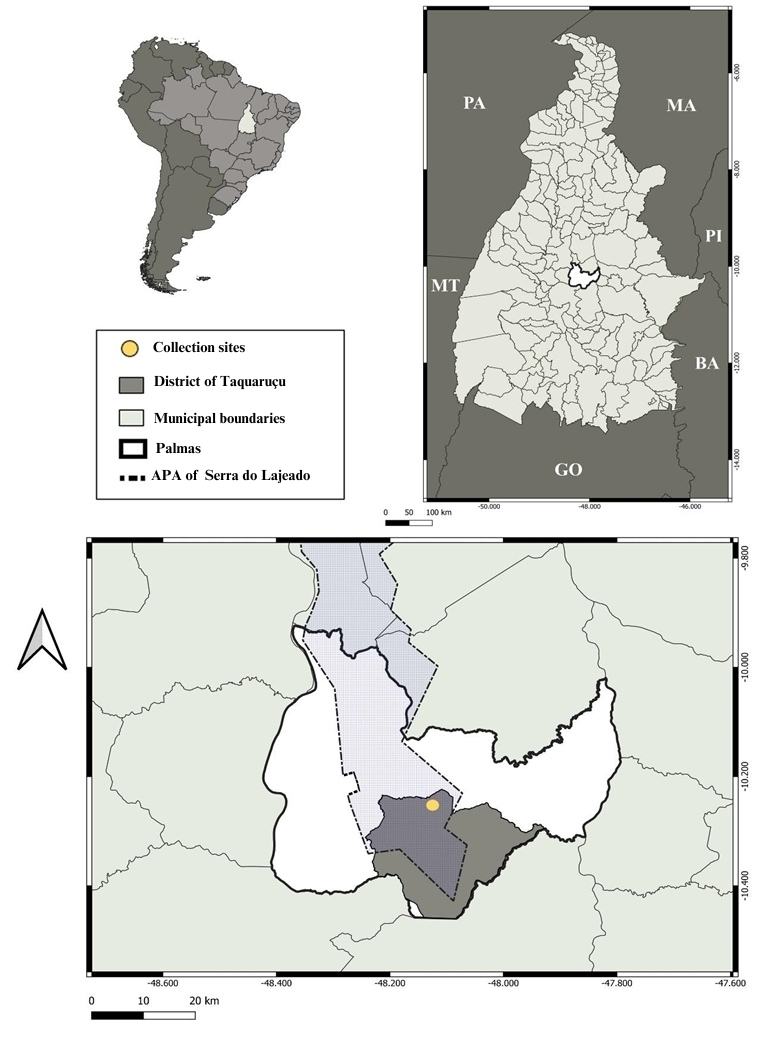
Location maps of the study area showing the state of Tocantins, municipality of Palmas, district of Taquaruçu and collection site (yellow dot) at Hotel Fazenda Encantada.

Located in the northern region of the country, the state of Tocantins is a transition region between the Cerrado and the Amazon biomes, with 72% of its entire territory formed by the Cerrado and 28% by the Amazon forest (Brasil, 2015). The study area is located within Serra do Lajeado Environmental Protection Area (APASL) at Hotel Fazenda Encantada (10°14’48.80”S; 48° 7’22.78”W), located 29 km from the state capital, Palmas (Figure 1).

There is wide altitude variation in the APASL, from 200 to 700 m.a.s.l., where the areas of lower altitudes form valleys, streams and rivers that are home to riparian and gallery forests. At higher altitudes, plateaus are formed on the top of the hills, where springs form streams that cascade down into waterfalls. The APASL also presents several phytophysiognomies of the Cerrado, such as Cerrado *stricto sensu*, Cerradão, Semideciduous Seasonal Forest, Gallery/Riparian Forests and Campos Cerrados (Naturatins, 2019).

### Material collection and identification

Tabanids were collected from horses, *Equus caballus* Linnaeus (Perissodactyla: Equidae). The horses chosen for the task had dark chestnut coats because tabanids seem to prefer dark horses (Bassi et al., 2000; Zamarchi et al., 2023). Horse flies were collected with an entomological net, then placed individually in deadly bottles containing ethyl acetate. Four campaigns were carried out, two in the dry season (June 16 and 17; September 22 and 23, 2012) and two in the rainy season (November 29 and 30, 2012; March 23 and 24, 2013), with a sampling effort of 13 hours per day (06:00h to 19:00h). The collections were carried out in the phytophysiognomy classified as typical Cerrado, which is characterized by predominantly tree-shrub vegetation, with tree cover of 20% to 50% with an average height of three to six meters (Ribeiro & Walter, 1998).

The identification of the material was based out by Lima et al. (2015). The specimens were deposited at the Federal University of Tocantins Entomology Collection (CEUFT) and Invertebrates Collection of the Instituto Nacional de Pesquisas da Amazônia (INPA), Manaus, Brazil

## Results

A total of 249 individuals were collected, classified into 25 species, 11 genera and two subfamilies (Pangoniinae and Tabaninae). *Fidena* (27.71%) was the most abundant genus, followed by *Stypommisa* (26.1%) and *Tabanus* (25.3%). A total of 53% of the flies were collected in the dry season and 47% were collected in the rainy season (Table 1).

**Table 1:** Absolute and relative abundances of Tabanidae species caught on horse in the district of Taquaruçu, municipality of Palmas, Tocantins state, Brazil.

**Species**	**Month**								**Total**	
	**June**		**September**		**November**		**March**			
	**n**	**%**	**n**	**%**	**n**	**%**	**n**	**%**	**n**	**%**
*Esenbeckia osornoi* Fairchild, 1942	10	4.0							10	4.0
*Fidena bistriga* Fairchild & Rafael, 1985					1	0.4			1	0.4
*F. castanea* (Perty, 1833)	6	2.4							6	2.4
*F. fumifera* (Walker, 1854)	6	2.4							6	2.4
*F. lissorhina* Gorayeb & Fairchild, 1987					56	22.5			56	22.5
*Catachlorops rufescens* (Fabricius, 1805)	16	6.4							16	6.4
*C. unicolor* (Lutz, 1912)	1	0.4							1	0.4
*Chlorotabanus inanis* (Fabricius, 1787)	1	0.4	2	0.8					3	1.2
*Dicladocera mutata* Fairchild, 1958			2	0.8					2	0.8
*Leucotabanus exaestuans* (Linnaeus, 1758)							3	1.2	3	1.2
*Philipotabanus henriquesi* Limeira-de-Oliveira, Gorayeb & Rafael, 2009					1	0.4			1	0.4
*Stypommisa aripuana* Fairchild & Wilkerson, 1986	47	18.9	17	6.8					64	25.7
*S. glandicolor* (Lutz, 1912)					1	0.4			1	0.4
*Poeciloderas quadripunctatus* (Fabricius, 1805)	3	1.2	1	0.4	4	1.6	8	3.2	16	6.4
*Tabanus antarcticus* Linnaeus, 1758	1	0.4			2	0.8	2	0.8	5	2.0
*T. cicur* Fairchild, 1942	1	0.4	2	0.8					3	1.2
*T. glaucus* Wiedemann, 1819					7	2.8			7	2.8
*T. importunus* Wiedemann, 1828			8	3.2					8	3.2
*T. mucronatus* Fairchild, 1961							3	1.2	3	1.2
*T. occidentalis* var. *consequa* Walker, 1850							3	1.2	3	1.2
*T. occidentalis* var. *dorsovittatus* Macquart, 1855	1	0.4	1	0.4	22	8.8	1	0.4	25	10.0
*T. occidentalis* var. *modestus* Wiedemann, 1828	4	1.61	1	0.4	2	0.8			7	2.8
*T. palpalis* Brèthes, 1910					1	0.4			1	0.4
*T. xuthopogon* Fairchild, 1984							1	0.4	1	0.4
Total	97	39.0	34	13.7	97	39.0	21	8.4	249	100

The most abundant species were *Stypommisa aripuana* Fairchild & Wilkerson, 1986 (25.7%), *Fidena lissorhina* Gorayeb & Fairchild, 1987 (22.49%), *Tabanus occidentalis* var. *dorsovittatus* Macquart, 1855 (10.04%), *Catachlorops rufescens* (Fabricius, 1805) (6.43%) and *Poeciloderas quadripunctatus* (Fabricius, 1805) (6.43%). Seven species had a relative abundance of less than 1%, representing only 3.21% of the total catch. The most abundant species represent 71.09% of the total tabanids collected (Table 1).

**Figure 2:**
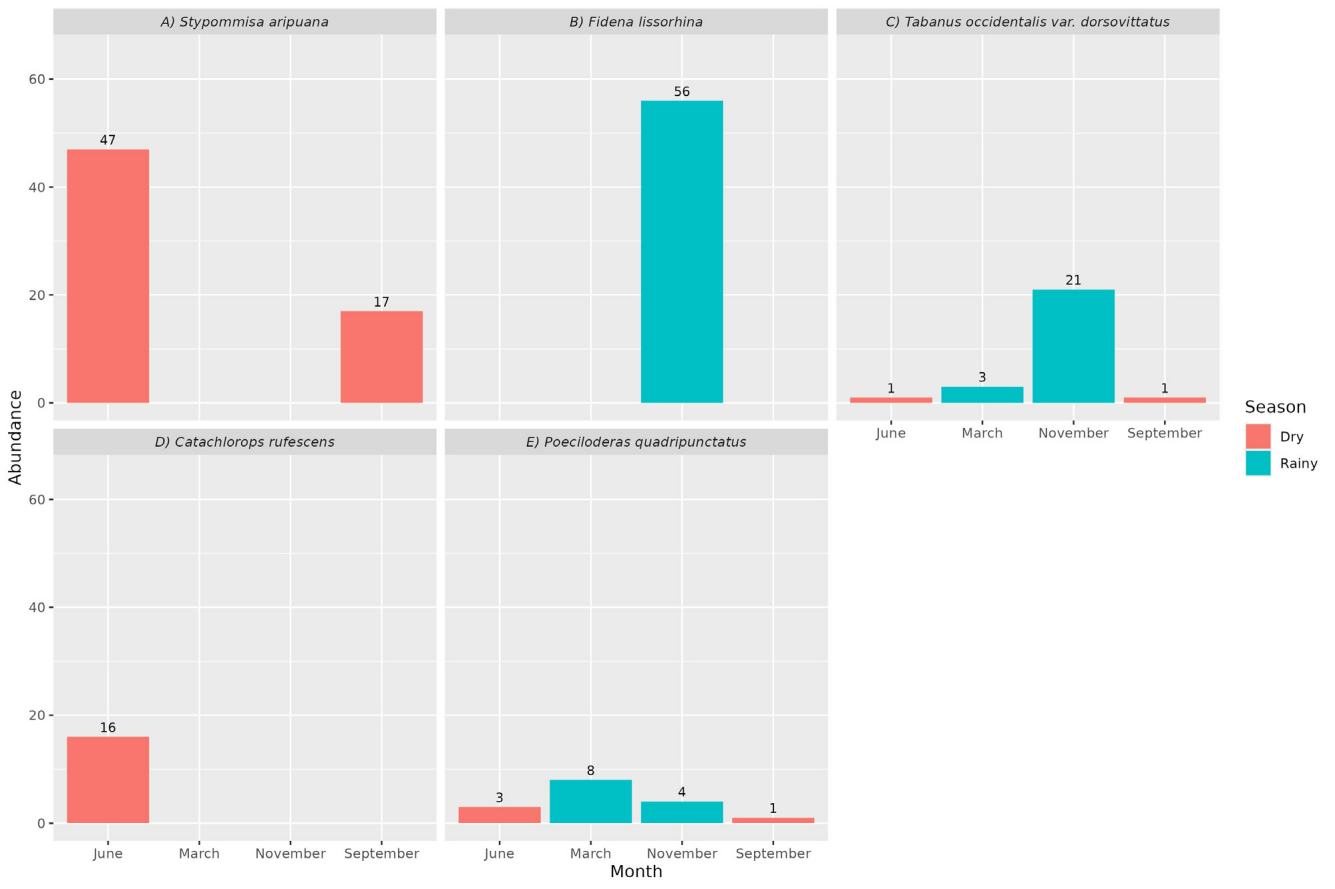
Abundance of the main Tabanidae species collected on horse in different seasons, in a cerrado area at the municipality of Palmas, Tocantins state, Brazil.

*Stypommisa aripuana* and *C. rufescens* were sampled only in the dry season, with the first occurring throughout the entire season but with a peak of abundance at the beginning of the season. In contrast, the second species was found only in the beginning that season (Figure 2A, 2D). *Fidena lissorhina* occurred only in the beginning of the rainy season (Figure 2B). *Tabanus occidentalis* var. *dorsovittatus* and *P. quadripunctatus* were sampled throughout the year but were more abundant in the rainy season. The first species was more frequently collected at the onset of the rainy season in November, and the second in the end of March (Figure 2C, 2E).

**Figure 3:**
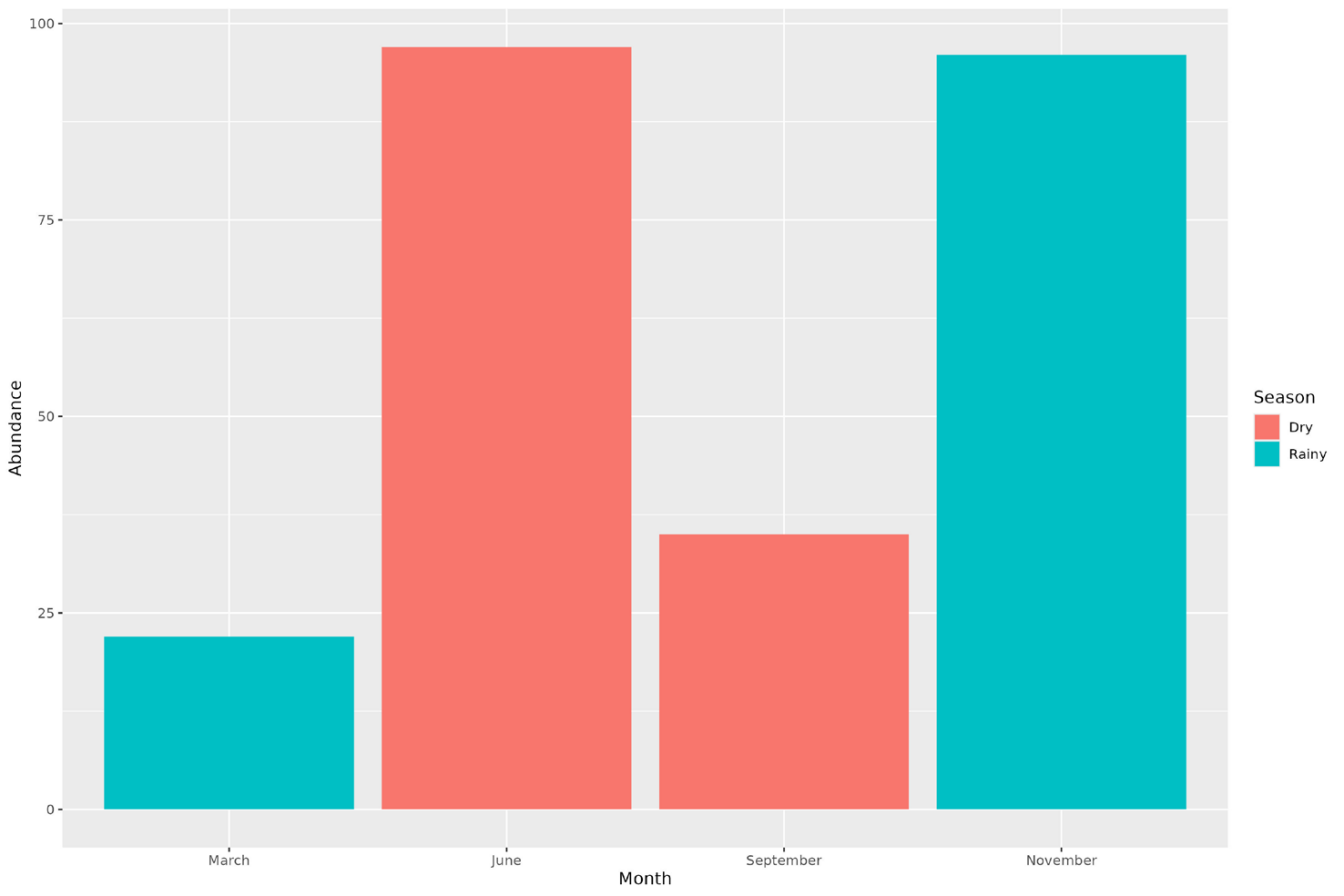
Abundance of Tabanidae collected on horse in different seasons in a cerrado area at the municipality of Palmas, Tocantins state, Brazil.

Although relatively close, the numbers of flies in the dry season (n= 131) exceeded the numbers in the wet season (n= 118). The number of tabanids was greater at the beginning of each season, collection peaks in June (dry season) and November (rainy season). At the end of each season (September and March), the number of tabanids collected gradually decreased (Figure 3).

## Discussion

The results allowed the identification of three occurrence patterns of the most frequent species, all of which may have implications for transmitting pathogens to horses at different times of the year in the cerrado of Tocantins. The first pattern concerns the occurrence of a species in a defined station. The adults of *F. lissorhina* only in the rainy season and of *S. aripuana* and *C. rufescens* only in the dry season. The second pattern concerns the occurrence of a species in both seasons. The pattern shows that *T. occidentalis* var. *dorsovittatus* and *P. quadripunctatus* occur both in the dry and rainy seasons, but more frequently in the latter. The third pattern presents species that occur at the beginning of the season and others that occur at the end of the season. The species *F. lissorhina* and *T. occidentalis* with greater numbers at the beginning of the rainy season, while high numbers of *P. quadripunctatus* occur toward the end.

Fairchild (1942) observed two patterns in Panama, classifying horse fly species into three groups. First, there are horse flies that do not have a defined flight season. Second, there are horse flies that are more numerous in one of the seasons, usually the rainy season, as observed for *T. occidentalis* and *P. quadripunctatus*; and finally, there are those horse flies that have a well-defined flight season, either in the rainy or dry season. The first pattern is represented by *F. lissorhina* and *S. aripuana*, the most frequent in different seasons of the year. They should be given more attention regarding the transmission of pathogens in the rainy and dry seasons, mainly in the beginning of each season. As observed in the present study, *S. aripuana* was among the most abundant species in Western Amazon (Zamarchi et al., 2023). It was collected only in August and September, the driest months in Rondônia. That species has a restricted distribution in the states of Mato Grosso, Tocantins and Rondônia (Coscarón & Papavero, 2009) and has only been registered in the last two states in the last nine years (Lima et al., 2015; Zamarchi et al., 2023). Other *Stypommisa* species were also collected only in the dry season, in Central Amazonia (Ferreira-Keppler et al., 2010) and the pantanal-cerrado ecotone (Koller et al., 2019); however, *S. captiroptera* was collected throughout the rainy season in eastern Amazonia (Gorayeb, 1993).

Until now, the distribution of *F. lissorhina* remains restricted to the states of Pará and Tocantins (Coscarón & Papavero, 2009), being collected from hosts in the rainy season (Gorayeb & Fairchild, 1987; Lima et al., 2015). Another two species of *Fidena* (5%), *F. fumifera* and *F. castanea*, contrary to *F. lissorhina*, occurred only in the dry season.

Even at the beginning of the dry season, *S. aripuana*, *C. rufescens* and *P. quadripunctatus*, which were abundant, with emphasis on the first two. In contrast, even with a reduction in total number at the end of this season, *S. aripuana* and *P. quadripunctatus* persisted in the environment. The beginning of the rainy season was marked by the elevated prevalence of *F. lissorhina* and *T. occidentalis* var. *dorsovittatus* and by the increase in the numbers of *P. quadripunctatus* during the dry season. At the end of the rainy season, *F. lissorhina* was no longer observed. There was a significant reduction in the numbers of *T. occidentalis*, with a predominance of *P. quadripunctatus*.

*Catachlorop rufescens* is distributed in the states of Roraima, Pará, Amazonas, Maranhão, Tocantins, Mato Grosso and Rondônia (Coscarón & Papavero, 2009), with captures on horses carried out in the states of Rondônia (Zamarchi et al., 2023), Pará (Gorayeb, 1993) and Tocantins (Lima et al., 2015). In Rondônia, the species was not collected on horse and, in Pará, its relative abundance on this host was less than 1%, wich is lower than the 6% observed in this study.

*Poeciloderas quadripunctatus* is distributed throughout the Neotropics (Coscarón & Papavero, 2009) and has been observed in association with horses in the Brazilian states of Santa Catarina (Miletti et al., 2011), Rondônia (Zamarchi et al., 2023), Pará (Gorayeb, 1993) and Amazonas (Rafael & Charlwood, 1980; Rafael, 1982). In these states, this species was observed in low numbers, usually less than 1%, except for the study carried out in Planalto Serrano de Santa Catarina (Miletti et al., 2011), where it reached 2.4%. Adults of this species seem to have a preference to fly in seasons with higher rainfall (52%) (Miletti et al., 2011), as observed in this study in the cerrado biome of Tocantins.

Tabanids have great potential for mechanical transmission of pathogens and have been associated with disease outbreaks that affect livestock. Some species are more abundant and efficient vectors, such as those in the genus *Tabanus*, which have been considered as the primary vector species of pathogens within the Tabanidae family (Silva et al., 2002; Parra-Henao et al., 2008; Silva, 2006). In the biome cerrado of Tocantins, this genus was abundant, representing the second most sampled genus (25.3% of the total).

In the Pantanal, *T. importunus* was one of the most prevalent flies in the rainy season, being considered as one of the main vectors of *Trypanosoma evansi*. The peak of this fly coincided with a major prevalence of trypanosomiasis in the region (Silva et al., 1995). In the biome cerrado of Tocantins, this species represented only 3% of the total sample being collected only in the dry season. The most prevalent species of *Tabanus* in the Cerrado of Tocantins was *T. occidentalis* var. *dorsovittatus* (10.04%), which peaked in the rainy season. In the Pantanal biome, this species represented 8% of horse flies collected, with activity peaks in the beginning of the rainy season. In the Eastern Amazon, it was the most prevalent species in pasture (61.7%) (Gorayeb, 1993, 2000) and the species that carried the greatest number of bacteria (Luz-Alves et al., 2007). In both Amazon studies the peak of this species contrasted with the collections from the Cerrado and the Pantanal which occurred at the beginning of the dry season.

Most species of Tabanidae from the Cerrado of Tocantins were relatively little abundant. The seven species representing 1% of the samples correspond to 3.21% of all individuals, and only five species represent 71.09% of the total number of tabanids collected in the study. Such high species richness with a low relative abundance of most species has been observed in other biomes (Gorayeb, 1993; Barros, 2001; Bassi et al., 2000; Krüger & Krolow, 2015; Zamarchi et al., 2023).

Tabanids are active year-round in tropical regions, but their peaks depend on the season and latitude, as with other insects (Wolda, 1988). In areas of higher latitudes in subtropical climates, such as in southern Brazil and Uruguay, the high prevalence of tabanids is associated with the warmest months of the year, as in the highlands of Santa Catarina (Miletti et al., 2011), eastern Pampa of Rio Grande do Sul (Krüger & Krolow, 2015) and the Uruguayan Pampa (Lucas et al., 2020). In the winter, when the temperatures decrease, the number of tabanids also decrease. In the biome cerrado of Tocantins, a tropical region, the number of horse flies caught on a horse was similar in both seasons. Tabanids were abundant during the dry season, with a collection peak at the beginning of the rainy season; the activity decreased only at the end of the rainy season. In eastern Amazonia, tabanid flies also showed activity throughout the year, with collection peaks throughout the dry season and at the beginning of the rainy season (Gorayeb, 1993). The seasonality of the Amazon contrasts with that of the Pantanal, where the greater numbers occur at the end of the dry season, extending throughout the rainy season (Barros, 2001). The same pattern as in the Pantanal was observed for a Pantanal-Cerrado ecotone region (Koller et al., 2019).

Horseflies have all the characteristics of suitable mechanical vectors. They feed intermittently, are highly mobile and have large mouthparts proportional to their body size (Foil, 1989). The large amount of blood they need to maturate their eggs (anautogeny), associated a blood meal by cutting the host's skin (telmophagy), causes pain and discomfort. These characteristics may induce host reactions that lead to interruption of feeding, with tabanids eventually returning to the same host (Barros & Foil, 2007). Therefore, the high abundance with which some species occur in the environment increases the probability of that species being involved in the mechanical transmission of pathogens (Barros & Foil, 1999). In addition to abundance, several other factors influence the mechanical transmission by tabanids; however, in the absence of more specific information, abundance may be a preliminary indicator of the potential importance of a species.

The direct influence of vector abundance on pathogen transmission highlights its role in increasing the likelihood of diseases spreading to humans or animals (ECDC, 2018; Cator et al., 2020; Wilson et al., 2020). With more abundant vectors, pathogens find efficient carriers, moving more frequently between hosts and raising the chances of establishing within populations. Seasonal fluctuations in insect population tend to impact disease dynamics, as population peaks have been sometimes correlated with disease outbreaks. The persistence of pathogens in the environment is closely tied to vector abundance, which can maintain disease circulation even outside of outbreak periods. Therefore, targeting control measures and allocating resources effectively requires a nuanced understanding of vector abundance and its epidemiological implications, emphasising the need for focused monitoring and intervention strategies for different vector groups based on their impact on livestock.
